# The Role of Oxidative Stress in Acute Ischemic Stroke-Related Thrombosis

**DOI:** 10.1155/2022/8418820

**Published:** 2022-11-16

**Authors:** Zhifang Li, Rentang Bi, Shuai Sun, Shengcai Chen, Jiefang Chen, Bo Hu, Huijuan Jin

**Affiliations:** Department of Neurology, Union Hospital, Tongji Medical College, Huazhong University of Science and Technology, Wuhan 430022, China

## Abstract

Acute ischemic stroke is a serious life-threatening disease that affects almost 600 million people each year throughout the world with a mortality of more than 10%, while two-thirds of survivors remain disabled. However, the available treatments for ischemic stroke are still limited to thrombolysis and/or mechanical thrombectomy, and there is an urgent need for developing new therapeutic target. Recently, intravascular oxidative stress, derived from endothelial cells, platelets, and leukocytes, has been found to be tightly associated with stroke-related thrombosis. It not only promotes primary thrombus formation by damaging endothelial cells and platelets but also affects thrombus maturation and stability by modifying fibrin components. Thus, oxidative stress is expected to be a novel target for the prevention and treatment of ischemic stroke. In this review, we first discuss the mechanisms by which oxidative stress promotes stroke-related thrombosis, then summarize the oxidative stress biomarkers of stroke-related thrombosis, and finally put forward an antithrombotic therapy targeting oxidative stress in ischemic stroke.

## 1. Introduction

Over the past few decades, stroke has become a leading threat to the health of people worldwide with a mortality rate of about 157 per 100,000 people, among which ischemic stroke accounts for more than 70% of all strokes [1, 2]. High incidence, disability, and recurrence rate of ischemic stroke have caused a heavy burden on the society. Currently, intravenous thrombolysis, arterial thrombolysis, and mechanical thrombectomy are the only available methods for acute recanalization for ischemic stroke [3]. However, only less than 5% of ischemic stroke patients can receive recanalization treatment due to the limitation of the treatment time window [3]. Even among patients who have received recanalization treatment, more than half of them do not benefit from it, and they even experience aggravation or death [4, 5]. Thus, there is an urgent need to identify more effective therapies for the prevention and treatment of ischemic stroke.

Encouragingly, progress has been made in the research of ischemic stroke in the recent years and neuroscientists have shifted their attention to oxidative stress in stroke-related thrombus formation [6, 7]. Oxidative stress refers to a harmful state in which the body produces numerous free radicals including reactive oxygen species (ROS) and reactive nitrogen species (RNS) [8]. In patients with high risk of ischemic stroke, oxidative stress is often triggered in advance. In atherosclerotic ischemic stroke, the occurrence of endothelial and macrophage oxidative stress is associated with atherosclerotic plaque progression [9, 10]. For cardiogenic stroke, basic cardiovascular diseases including cardiac insufficiency and arrhythmia have long been proven to be accompanied with significant oxidative stress [11, 12]. Once initiated, oxidative stress can promote thrombus formation by inducing endothelial dysfunction, promoting platelet activation, facilitating platelet-leukocyte aggregation, and modifying fibrinogen function [13–16]. More importantly, oxidative stress has an impact on the maturation and stability of stroke-related thrombus, which might make it more difficult to be dissolved by conventional thrombolytic drugs and worsen the functional outcome of these patients [17]. Taken together, targeting oxidative stress might be a promising method for the prevention and thrombolytic treatment of stroke-related thrombus formation.

In this review, we are particularly concerned with the processes during which oxidative stress in the blood vessels leads to thrombosis and ischemic stroke. Firstly, we discuss the mechanisms by which oxidative stress promotes the formation of stroke-related thrombus. Then, we summarize the oxidative stress biomarkers of stroke-related thrombosis. Finally, we put forward antithrombotic therapies targeting oxidative stress in ischemic stroke.

## 2. Generation of Oxidative Species and Antioxidants in Ischemic Stroke

### 2.1. Oxidative Stress and Reactive Species

Oxidative stress basically refers to the disturbances in the prooxidant-antioxidant balance, resulting in the generation of ROS and RNS [18].

ROS comprise several oxygen intermediates, mainly including superoxide anion (O_2_^−^), hydrogen peroxide (H_2_O_2_), peroxyl radicals (HO_2_), and hydroxyl radicals (OH), among which O_2_^−^ seems to be the primary product of ROS [19]. After its production, O_2_^−^ generates H_2_O_2_ with the help of superoxide dismutase (SOD) or the more reactive HO_2_. Then, H_2_O_2_ interacts with ferrous iron which is reduced by O_2_ to produce OH [20, 21].

RNS mainly include two species, nitric oxide (NO) and peroxynitrite anion (ONOO-). NO is produced through the enzymatic reaction of L-arginine and O_2_, which is catalyzed by three types of NO synthases: inducible NO synthase (iNOS), endothelial NO synthase (eNOS), or neuronal NO synthase (nNOS). Among these three types, iNOS and nNOS are considered to be harmful isoforms in ischemic stroke [22, 23], while eNOS seems to be protective. In the study by Huang et al., eNOS^−/−^ mice showed increased infarction volume compared to wild-type mice [24]. The increased NO in turn prefers to interact with O_2_^−^ to generate ONOO-, which has a stronger oxidation capacity than NO or O_2_^−^ alone [25].

### 2.2. The Mechanism of Oxidative Species Generation in Stroke-Related Thrombosis

The source of oxidative stress is very complex, and currently the following pathways have been implicated in its production: decoupling of the mitochondrial respiratory chain, and activation of xanthine oxidase (XO) and nicotinamide adenine dinucleotide phosphate (NADPH) oxidases (NOX). Under physical conditions, the mitochondrial respiratory chain is able to use oxygen to generate energy. However, several circumstances, such as ischemia, can lead to inhibition of the mitochondrial respiratory chain at complex IV, and then the intermediates produced by complex I-III accumulate and interact with O_2_ to produce ROS [26]. In addition, activation of XO also contributes to ROS generation. In normal conditions, XO serves as an exchangeable form of xanthine hydrogenase (XDH). When intracellular ATP is depleted, it can lead to the accumulation of compounds, such as xanthine and hypoxanthine, which are substrates of XO. Then, XDH can be cleaved into active XO to eliminate them, generating ROS simultaneously [26]. Finally, NOX plays an important role in ROS production [26]. There are seven members of the NOX family (NOX1, NOX2, NOX3, NOX4, NOX5, DUOX1, and DUOX2), among which the NOX4 subtype is expressed most abundantly and considered as the most relevant source of ROS in the central nervous system [27]. Normally, NOX can reduce one electron from O_2_ to produce O_2_^−^ using NADPH as an electron donor, while pathological conditions can cause O_2_^−^ overproduction by NOX.

Additionally, increasing attention has been paid to the potential role of myeloperoxidase (MPO) as another source of oxidative stress. Typically, MPO is mainly expressed in the granules of quiescent neutrophils and remains inactive in the absence of H_2_O_2_ [28]. Upon neutrophil activation, NOX2 can be recruited to the internal membrane of neutrophils, which subsequently leads to the burst of oxidative stress and production of H_2_O_2_ [29]. Once the level of H_2_O_2_ is increased in neutrophils, MPO is able to catalyze a series of one- or two-electron oxidative reactions and transiently produce multiple redox heme-iron species. The halogenation cycle is the first step in the catalytic chain of MPO, which involves the conversion of the native (Fe(III))-MPO into the reactive “Fe(IV) = O ⋯ por•+” (MPO compound I). Compound I can then oxidize halide anions (Br-, Cl-, and SCN-) to form multiple hypohalous acids (HOBr, HOCl, and HOSCN) [30]. Of note, HOCl and HOSCN are considered as the key MPO-derived oxidative stress species in endothelial dysfunction and stroke-related thrombosis. Subsequent to the halogenation cycle is the peroxidase cycle, during which compound I can oxidize various small molecule substrates including nitrite, NO, and H_2_O_2,_ and form diffusible substrate radicals (RH•) with the help of compound II (Fe(IV) = O) [31].

Besides the classic pathways, novel mechanisms involved in oxidative species generation have also been identified, and they include ferroptosis. Ferroptosis is defined as an iron-dependent form of cell death due to the accumulation of lipid hydroperoxides [32]. It is a newly discovered mechanism of cell death, which is different from apoptosis, necrosis, and autophagy. The main biological characteristics of ferroptosis include the following: (1) overload of iron ions, (2) depletion of glutathione (GSH) and inactivation of glutathione peroxidase-4 (GPx-4), and (3) accumulation of iron-dependent lipid hydroperoxides [33]. In response to pathological conditions, lipoxygenases can oxidize polyunsaturated fatty acids (PUFAs) to generate ROS. When iron metabolic dysfunction causes iron accumulation in the cytoplasm, lipid hydroperoxides can be further converted into toxic lipid free radicals [34]. In addition to iron-mediated ROS production, GPx-4 inactivation due to depletion of GSH during ferroptosis can also induce the production of ROS from lipid peroxidation [35].

In addition to ferroptosis, it is interesting to note that endoplasmic reticulum (ER) stress may also be involved in the production of reactive species. ER stress is defined as a process in which the accumulated unfolded or misfolded proteins cause a stress condition in the ER, which in turn activates unfolded protein response (UPR) proteins to initiate adaptive signaling events. Once activated, the ER can provide a unique environment which favors protein folding and promotes the formation of disulfide bonds to produce ROS [36]. Appropriate ER stress may be helpful to restore ER homeostasis, while prolonged ER stress can result in the accumulation of ROS, exacerbating the oxidative stress. The most studied route of ROS generation by ER stress is the protein disulfide isomerase (PDI) and ER oxidoreduction 1 (ERO1) pathway. To be specific, PDI can oxidize thiols in folding substrates to form disulfide bonds, leaving PDI in a reduced state. Then, ERO1 can reoxidize reduced PDI through a flavin adenine dinucleotide (FAD)-dependent reaction, resulting in H_2_O_2_ formation [37]. Additionally, NOX4 also participates in the production of superoxide anion and hydrogen peroxide in the ER transmembrane [38].

### 2.3. Antioxidant Mechanisms

To prevent excessive oxidative stress-induced damage to the body, there are endogenous and exogenous antioxidant mechanisms against oxidative stress. Endogenous mechanisms comprise enzymatic and nonenzymatic pathways; and SOD, catalase (CAT), and GPx play crucial roles in the enzymatic pathway. Specifically, SOD can react with O_2_ and convert it into H_2_O_2_, which is subsequently decomposed by CAT into H_2_O and O_2_ [39], thus preventing the production of hydroxyl radicals. In addition, GPx can reduce H_2_O_2_ into H_2_O by oxidizing GSH into glutathione disulfide [40]. In the nonenzymatic pathway, antioxidants can directly bind to transition metal ions, such as iron or copper, thus terminating the free radical chain reactions [41]. This is achieved mainly by bilirubin, *α*-tocopherol (vitamin E), *β*-carotene, albumin, and uric acid (UA) [42]. Finally, exogenous antioxidants are not produced in the human body, but they are obtained from food or drug intake, such as ascorbic acid (vitamin C), phenolic antioxidants (phenolic acids, resveratrol, and flavonoids), oil lecitinas, and selenium [43].

## 3. The Mechanism of Oxidative Stress in Stroke-Related Thrombosis

Generally, it is believed that the following two interdependent mechanisms are involved in the process of thrombus formation: platelets and coagulation factors pathway. Under the circumstance of endothelial injury, platelets will firstly adhere to the exposed subendothelial matrix with the help of von Willebrand factor (vWF) [44] and become activated when they interact with collagen [45]. Then, the activated platelets will secrete multiple proaggregatory substances, such as adenosine diphosphate (ADP) and thromboxane A2 (TXA2), which further facilitate platelet aggregation, adhesion, and activation [46]. At the same time, the coagulation cascade is initiated when the injured vessel releases tissue factor, the primary trigger of the extrinsic coagulation process, into the blood [47]. Following its release, a number of coagulation factors (FVII, FX, FIX, and FII) are activated, ultimately leading to the formation of fibrin, which accounts for the highest proportion of protein in the thrombus. In addition, there is growing evidence that leukocytes, especially neutrophils, play an essential role in thrombus formation. Leukocytes not only secrete soluble compounds to active platelets but also form heteroaggregation with platelets, known as platelet-leucocyte aggregates [48, 49]. Moreover, the tissue factor can also be released by activated platelets and further promotes fibrin formation [50].

**Figure 1:**
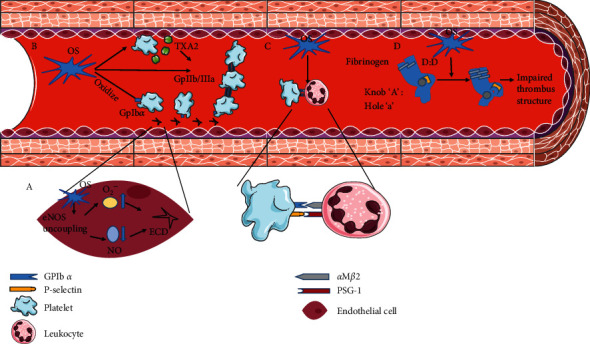
The mechanism of oxidative stress in stroke-related thrombosis. (a) Oxidative stress and endothelial dysfunction. (b) Oxidative stress and platelet function. (c) Oxidative stress and platelet-leukocyte aggregation. (d) Oxidative stress and fibrinogen modification.

In recent years, it has been found that oxidative stress also participates in the process of thrombosis and influences the biochemical properties of thrombus by interacting with the above pathway [6]. Several oxidative stress biomarkers have been found to predict the risk of stroke-related thrombosis [51]. Thus, thorough understanding of the underlying mechanisms may greatly contribute to novel antithrombotic treatments for ischemic stroke. In the following sections, we will elucidate the detailed mechanisms based on the following aspects: endothelial dysfunction, platelet function, platelet-leukocyte aggregation, and fibrinogen modification (Figure 1).

### 3.1. Oxidative Stress and Endothelial Dysfunction

Under physical conditions, the functional endothelial cells in the vasculature are essential for maintaining the balance between fibrinolysis and thrombosis, vasodilation and vasoconstriction, promotion and inhibition of smooth muscle cell proliferation, and adhesion molecules expression [52, 53]. However, in response to various stimuli, such as oxidative stress, endothelial dysfunction will occur, which is regarded as the earliest phenotypic alteration in the vasculature leading to thrombosis in ischemic stroke [15]. Endothelial dysfunction is defined as a shift from the normal endothelial phenotype to the one that promotes thrombosis, vasoconstriction, smooth muscle cell proliferation, and circulating leukocyte adherence [54].

Mechanically, the decreased synthesis and activation of NO are considered as one of the earliest and most important events that initiate endothelial dysfunction and thrombosis [55]. As previously mentioned, NO plays a crucial role in mediating normal endothelial function [56, 57]. Among the three NOS isotypes (iNOS, eNOS, and nNOS), which are involved in NO synthesis, eNOS is the most abundantly expressed in the endothelium and is regarded as a key determinant for maintaining normal endothelial function [58, 59]. Under physiological conditions, eNOS can use O_2_ and L-arginine as substrates to catalyze the production of NO with the help of the cofactor tetrahydrobiopterin (BH4). However, BH4 can be oxidized to BH2 by O_2_^−^ or ONOO- under oxidative stress, which then results in “uncoupling” of eNOS [60]. Subsequently, NO generation is interrupted and eNOS starts to produce superoxide O_2_^−^ instead of NO, thus disrupting the endothelial function. Moreover, superoxide O_2_^−^ synthesized by eNOS can further promote the oxidation of BH4 to BH2, exacerbating this state [61]. Thus, oxidation of BH4 by ROS seems to represent a major mechanism that explains eNOS uncoupling. Interestingly, treatment with BH4 supplementation showed reduced NOS-dependent generation of superoxide [62] and restored endothelial function [63, 64], indicating that BH4 may be an ideal target for oxidative stress-induced “uncoupling” and endothelial dysfunction.

Additionally, there is mounting evidence suggesting that vascular NOX also participates in the phenomenon of eNOS uncoupling. An important clue was obtained from the study by Landmesser et al., which used NOX p47phox knockout mice [60]. They found that there was markedly increased ROS production by eNOS uncoupling in a mouse model of hypertension and NOS inhibitor L-NAME could abrogate the ROS elevation. However, p47phox knockout hypertensive mice showed lesser ROS production and L-NAME could no longer reduce the ROS level [60]. From this research, it can be inferred that oxidative stress generated by NOX may also induce eNOS uncoupling to cause endothelial dysfunction and subsequent stroke-related thrombosis.

In addition to eNOS uncoupling, clinical studies have also confirmed that the serum MPO level is inversely associated with brachial artery flow-mediated dilation which serves as an indicator of endothelial function in human subjects [65]. This result highlighted the potential role of MPO in the pathogenesis of endothelial dysfunction. Currently, a large number of studies are investigating the mechanisms by which MPO-generated products can deteriorate the endothelial function. First of all, MPO is able to oxidatively suppress the kallikrein-kinin system, and results in reduced generation of bradykinin, also known as eNOS agonist [66]. As mentioned above, MPO is able to interact with a wide range of substances producing oxidative species, which may influence endothelial dysfunction. Among these oxidative species, HOCl and HOSCN are considered as the most significant factors and both of them can directly oxidize and depolymerize eNOS leading to eNOS uncoupling [67, 68]. Moreover, HOCl is capable of chlorinating L-arginine, the eNOS substrate, to produce chlorinated arginine, which is the competitive inhibitor of eNOS [69]. In addition, HOCl- or HOSCN-modified low-density lipoprotein (LDL) may also contribute to endothelial dysfunction by causing eNOS uncoupling [70]. Finally, HOCl and HOSCN can increase the expression of tissue factor in endothelial cells and adhesion molecules in neutrophils, contributing to redox disturbance [71].

Oxidative stress can produce excessive superoxide, which in turn interacts with NO to generate OONO-. Excessive OONO-generation has also been found to induce protein nitration and cause endothelial dysfunction in human umbilical vein endothelial cells *in vitro* [72]. On the one hand, the reaction with superoxide can directly decrease the amount of NO in the endothelium. On the other hand, OONO- can lead to the inactivation of NO and contribute to the oxidation of BH4 [73, 74], further exacerbating eNOS uncoupling. XO, one of the key enzymes involved in ROS generation, also participates in endothelial dysfunction-induced thrombosis. In an animal model of paroxysmal atrial fibrillation, inhibition of XO by febuxostat significantly attenuated endothelial dysfunction and decreased the thrombogenesis risk in the left atrium [75]. On account of the close association between atrial fibrillation and cardioembolic stroke, XO may also contribute to the formation of stroke thrombus. Moreover, PDI, the key enzyme in ER-induced ROS production, can be secreted by endothelial cells upon vascular injury. The released PDI can then activate a large number of extracellular substrates, such as platelet factor V in the vasculature, leading to the formation of thrombus [76, 77]. However, the role of PDI in stroke-related thrombosis has not been investigated so far and further studies are needed. Another possible mechanism of oxidative stress-induced endothelial dysfunction involves the antioxidant enzyme GPx-4 and vitamin E. In mice with combined deficiency of GPx-4 and vitamin E, endothelial dysfunction occurred and endothelial cells were detached from the basement membrane, resulting in multiorgan thrombus formation including the brain vessels [78].

### 3.2. Oxidative Stress and Platelet Function

It is well known that platelets play a crucial role in mediating hemostasis and thrombosis. After vascular injury, platelet adhesion to the endothelium occurs rapidly, by means of the interaction between platelets and activated endothelial cells via receptor-ligand binding [79]. Once adhered to the endothelial cells, platelets are activated by upregulated integrins and release substances such as thromboxane A2 (TXA2) and adenosine diphosphate (ADP) to recruit other resting platelets [13]. Finally, increased number of platelets aggregate through the activation of glycoprotein IIb/IIIa (GpIIb/IIIa) receptors to promote thrombosis [80].

Currently, a growing number of studies are investigating the roles of ROS/RNS in platelet function and stroke-related thrombosis [81]. Upon platelets activation, enzymes involved in platelet ROS production are motivated and platelets can then produce free radicals [81]. Among all potential sources of ROS, NOX seems to be the most important one involved in platelet function, and suppression of NOX has been found to significantly inhibit platelet aggregation and thrombus formation *in vitro* under high shear conditions [82]. Moreover, in patients with advanced atherosclerosis, NOX inhibitor apocynin can reduce platelet adhesion [83]. NOX has several isoforms; NOX2 is abundantly expressed in platelets and generally regarded as the most relevant source of ROS in platelets [84]. This is further verified by a study suggesting that almost completely impaired ROS generation from platelets occurs in patients with hereditary deficiency of NOX2 [85]. Moreover, in an *in vitro* experiment of anoxia-reoxygenation, NOX2 has been found to upregulate platelet ROS, which ensues the secretion of TXA2 and isoprostanes, leading to platelet activation [86]. From these studies, it can be inferred that NOX2 may be implicated in the functional changes in platelets and may in turn promote ischemic stroke-related thrombosis. Additionally, a role for PDI, the key enzyme involved in ER-associated oxidative stress production, has also been observed in platelet function modulation. In mice with platelet-specific deficiency of PDI, platelets showed significantly defective aggregation when stimulated with several agonists, and further intravital microscopy analysis showed that PDI was responsible for subsequent platelet accumulation but not for initial platelet adhesion [87].

Besides the enzymes involved in platelet ROS production, ROS may also directly affect platelet function in stroke-related thrombosis. In platelets, ROS including O_2_^−^, OH, and H_2_O_2_ act as second messengers to influence platelet function through calcium mobilization, NO inactivation, and isoprostane formation [6]. Firstly, platelet-derived ROS have been shown to oxidize sulfhydryl groups of GpIb*α* in the platelet membrane, which in turn enhances their adhesion to endothelial cells by interacting with vWF [14]. This suggests that ROS participates in the initial stage of thrombosis in ischemic stroke by promoting platelet adhesion. Secondly, ROS is associated with platelet activation and granular molecule release in ischemic stroke-related thrombosis. NOX-derived O_2_^−^ can lead to increased release of TXA2 from platelets via the PLA2-dependent mechanism [88]. In addition, H_2_O_2_ can directly cause calcium mobilization within platelets, which subsequently induces TXA2 upregulation and release [89]. Finally, platelet recruitment is regulated by aggregating molecules such as GpIIb/IIIa and ROS seems to play a part in this process in ischemic stroke. It has been observed that H_2_O_2_ can lead to GpIIb/IIIa activation, which subsequently promotes platelet aggregation [90]. In contrast, ROS scavengers are found to inhibit platelet aggregation, while ROS donor, such as DNMQ, reverses this effect [82]. Additionally, it has been reported that ROS is able to promote the production of 8-iso-prostaglandin F2 alpha (8-iso-PGF2*α*), a reliable marker of oxidative stress and platelet function, which has been reported to contribute to platelet recruitment by activating GpIIb/IIIa [82]. Interestingly, in patients with X-linked chronic granulomatous disease (X-CGD), platelet production of 8-iso-PGF2*α* is decreased, which is associated with damaged platelet recruitment. However, pretreatment of platelets obtained from X-CGD patients with 8-iso-PGF2*α* significantly promotes platelet recruitment [82]. These results indicate that ROS-derived 8-iso-PGF2*α* also plays an essential role in platelet function and thrombosis development in ischemic stroke. Moreover, studies have suggested that MPO is another potent platelet stimulator, which is able to bind with platelets and subsequently activate them [91]. Of note, interaction with MPO can induce actin cytoskeleton reorganization within the platelets, increase its elasticity, and potentiate store-operated Ca2+ entry, all of which finally facilitate platelet aggregation [92]. From these studies, it can be concluded that MPO may be another potent platelet activator.

In contrast to ROS, RNS has been found to mainly suppress platelet function in ischemic stroke-related thrombosis. NO can inhibit the expression of platelet surface glycoproteins [93, 94], such as P-selectin and the integrin GpIIb/IIIa complex, which leads to impaired platelet adhesion as well as aggregation and prevents thrombosis [95]. Platelet aggregation can be promoted by incubation with inhibitors of eNOS, while incubation with the eNOS substrate, such as L-arginine, can reverse this phenomenon [96]. Mechanically, NO can lead to the activation of soluble guanylyl cyclase, which in turn increases the synthesis of cGMP. Subsequently, the platelets NO/cGMP signaling pathway can activate the vasodilator-stimulated phosphoprotein (VASP), whose phosphorylation (mostly at Ser 239) has been found to be a reliable biomarker for platelet function inhibition [97]. As mentioned previously, NO can interact with ROS to generate OONO-, and OONO- as well as its reactive intermediates are able to modify platelet functions [98]. A number of studies have shown that OONO- can inhibit platelet adhesion to collagen and fibrinogen [99], platelet activation, and release of ADP [100], as well as platelet aggregation [101]. The mechanism by which OONO- exerts its impact on platelets is complicated. OONO- can diffuse directly across the platelet membrane, resulting in lipid peroxidation [100] and oxidation or nitration of proteins [102, 103], which may contribute to the inhibited platelet function in ischemic stroke. However, the detailed mechanism remains elusive and further studies are required to answer this question.

Furthermore, the antioxidative system has also been linked to platelets function modulation and stroke-related thrombosis. In diabetes patients who are susceptible to thrombotic events, such as ischemic stroke, lower GSH, GPx, and SOD-1 levels are noted within platelets, while administration of N-acetylcysteine can reduce the risk of ischemic stroke via enhancing the platelet antioxidant enzyme activity [104]. GPx-3, a selenocysteine-containing antioxidant enzyme, has been found to inhibit platelet-dependent thrombosis [105]. In the GPx-3^−/−^ mice, ADP-induced platelet aggregation was more robust and the percentage of occluded vessels was higher compared with those in wild-type mice [106]. Moreover, a promoter haplotype of GPx-3 with reduced function is associated with increased stroke risk, indicating that normal GPx-3 function is crucial for preventing stroke, potentially by suppressing platelet-dependent thrombosis. In addition to GPx-3, the antioxidant enzyme GPx-1 also protects aged mice from platelet hyperactivity, which might increase the susceptibility to thrombosis [107].

### 3.3. Oxidative Stress and Platelet-Leukocyte Aggregation

Recent studies have provided convincing evidence that platelet-leukocyte aggregation on the activated endothelial cells plays a crucial role in thrombus formation in ischemic stroke [108, 109]. Once endothelial cells are inflamed and activated, neutrophils will adhere to the endothelium, which provides an adhesive surface that facilitates further platelet activation and aggregation. The platelet-leukocyte interactions mainly depend on the binding between platelet GpIb*α* and P-selectin, neutrophil *α*M*β*2 integrin and P-selectin glycoprotein ligand-1 (PSGL-1) [110]. Notably, it seems that the binding of GpIb*α* and *α*M*β*2 integrin is more indispensable for the stable attachment between platelets and neutrophils [111].

Oxidative stress is an important regulator of thrombus formation in ischemic stroke. However, it remains unknown whether oxidative stress can mediate platelet-leukocyte aggregation, which may induce stroke-related thrombosis. To answer this question, Bazzoni et al. firstly revealed that ROS released from leukocytes can induce platelet-leukocyte aggregation in an *in vitro* experiment [112]. Moreover, it seems that NOX2 in both platelets and neutrophils plays an important role in their interaction. Platelet NOX2 deletion was shown to significantly reduce platelet surface expression of P-selectin and impair ligand-binding activity of GpIb*α* during vascular inflammation [14]. Consistently, NOX2 knockout (KO) neutrophils also exhibited decreased activation and ligand-binding function of *α*M*β*2 integrin during vascular inflammation [14]. These results suggest that ROS generated by NOX2 from both neutrophils and platelets may be required for platelet-leukocyte interaction in ischemic stroke.

In contrast to ROS, NOS has been found to inhibit platelet-leukocyte aggregation [113] and improved endothelial NO bioavailability by treatment with prasugrel can reduce platelet-leukocyte interaction in patients with unstable angina pectoris [114]. Thus, NO may be a promising target to prevent platelet-leukocyte interaction and thrombus formation in ischemic stroke.

### 3.4. Oxidative Stress and Fibrinogen Modification

Fibrinogen is one of the most important components of the hemostatic system, which can be cleaved into fibrin by thrombin to promote thrombosis. Current studies have paid increasing attention to the posttranslational modifications of fibrinogen and its effect on clot formation. Notably, oxidative stress can interact with fibrinogen and several oxidative posttranslational modifications of fibrinogen have been found to influence stroke-related thrombosis.

In the study by Medeiros et al., they investigated the association between fibrinogen tyrosine nitration and the occurrence of ischemic stroke [16]. They found that three 3-nitrotyrosine residues (*β*Y452, *β*Y475, and *γ*Y380) showed significant differences between the ischemic stroke and control groups. Receiver operating characteristic (ROC) curve analysis further suggested that these 3-nitrotyrosine residues forms of fibrinogen can predict the occurrence of ischemic stroke individually or in combination [16]. From these results, it can be inferred that there is a close relationship between fibrinogen-bound 3-nitrotyrosine and stroke-related thrombosis, but the underlying mechanism remains elusive. A recent study further analyzed the influence of oxidative stress on fibrinogen function and fibrin network formation in patients with acute ischemic stroke [17]. The authors demonstrated that acute ischemic stroke patients showed a higher level of malondialdehyde, a marker of oxidative stress *in vivo*. Moreover, they found that the final optical density of clots was higher and the lysis time of clots was prolonged in acute ischemic stroke patients. Interestingly, the clots from these patients exhibited thicker fibers but lower fibers number in comparison with the control group [17]. Consistent with this finding, exposure of fibrinogen to oxidative stress such as H_2_O_2_ and NO *in vitro* also led to a similar change in the clot structure [115, 116]. These results highlighted that oxidative stress affects fibrin clot formation, structure, and dissolution, which may subsequently participate in stroke-related thrombosis and influence the effectiveness of thrombolytic therapy.

In the early stages of fibrin polymerization, the formation of knob 'A': hole 'a' and the D:D interaction between fibrinogen molecules are crucial. Shimizu et al. found that oxidative stress can lead to the destruction of the fibrin fragment desAB N-DSK (N-terminal disulphide knot region of fibrinogen lacking both FpA and FpB) on site A, rending it unable to bind to fibrinogen [117]. These results suggested that the knob 'A': hole 'a' interaction is impaired by oxidative stress, which may affect fibrin polymerization. In terms of the D:D interaction, Rosenfeld et al. revealed that D:D interaction between fibrins were enhanced when exposed to oxidative stress, which is responsible for assembling the cross-linked protofibrils from oxidized monomeric fibrin molecules [118]. The enhanced D:D interaction represents the essential adaptive molecular mechanisms in the oxidatively modified fibrin self-assembling processes.

After the initial stages of fibrin polymerization, subsequent polymerization processes include the formation of two-stranded protofibrils and three-dimensional network of fibrin [119]. In the research by Wang et al., they used UV absorbance spectroscopy, *ζ*-potential, dynamic light scattering, circular dichroism, and steady shear viscosity to investigate the effect of oxidative stress on these polymerization processes. They found that the branching, cross-linking, and height distribution of the formed fibrin were affected by oxidative stress, leading to the formation of amorphous fibrin and local bulk aggregation. Specifically, the altered equilateral junctions of protofibrils and cross-linking patterns between the *α*- and *γ*-chains were responsible for this occurrence [116]. Later, similar research also confirmed this conclusion [120]. Overall, the polymerization processes of fibrin are also influenced by oxidative stress, which may explain the structural alteration of fibrin observed in the clots of ischemic stroke.

## 4. Oxidative Stress Biomarkers in Stroke-Related Thrombosis

At present, early neurological progression and recurrence remain the major cause of poor prognosis in stroke patients [121, 122], while it is difficult to predict in clinical settings. Thus, the development of rapid and noninvasive tests would facilitate timely prediction and intervention, and laboratory biomarkers of stroke-related thrombosis have gained special attention. Progression and recurrence of stroke are associated with the progression of thrombus formation and arterial reocclusion, while oxidative stress plays a crucial role in the pathogenesis. For this reason, redox biomarkers with high specificity and easy detection characteristics may be important tools for predicting the occurrence of ischemic stroke and monitoring its progression and prognosis. Several studies have focused on identifying these redox biomarkers; and in the following sections, we have summarized these biomarkers based on the following four aspects: lipid peroxidation, DNA oxidation, antioxidants, and MPO.

### 4.1. Lipid Peroxidation Biomarkers

Lipid peroxidation products are not only widely used biomarkers of oxidative stress but also important triggering factors in stroke-related thrombosis. They are mainly derived from the PUFAs of oxidative stress species [123], and they participate in the process of ferroptosis to generate more toxic lipid free radicals. Initial lipid peroxidation results in the generation of conjugated dienic hydroperoxides, which are unstable and can be decomposed into various dienals, aldehydes, or alkanes. Among these products, malondialdehyde (MDA) and 4-hydroxy-2-nonenal (HNE) have been investigated in the most depth.

A large number of clinical studies have shown an increased concentration of MDA in both the serum and saliva of stroke patients, with a higher level in saliva than in serum [124, 125]. This suggests that increased MDA level may indicate the occurrence of thrombosis in stroke patients. Further analysis showed that both serum and salivary MDA can be ideal biomarkers for differentiating healthy individuals from ischemic stroke patients, with a higher accuracy rate of MDA in saliva (92%) than in serum (81%) [126]. Interestingly, patients with high risk of developing stroke-related thrombosis also exhibited a high level of MDA [126], which implies the possibility of using MDA to predict the risk of stroke and its progression. However, it should be noted that although MDA remains the most frequently used marker for lipid peroxidation, the measurement of MDA is actually not accurate. Currently, the measurement of MDA is based on the test of thiobarbituric acid (TBA), a substance which can react with various compounds in the body fluid [127]. Moreover, TBA itself can lead to the generation of extra MDA; therefore, an overestimation of the MDA level seems inevitable [127]. Thus, future studies need to innovate more accurate test methods, facilitating its role as a biomarker of stroke-related thrombosis.

In contrast to MDA, HNE can be detected through the way of immunological techniques with anti-HNE antibodies or high-performance liquid chromatography (HPLC) directly [128, 129]. It has been reported that plasma 4-HNE concentrations have been found increased in both experimental stroke rats and stroke patients [51]. Further study has also shown that there is a positive correlation between plasma HNE and homocysteine concentration, a known risk factor of thrombosis in stroke [51]. In conclusion, the results suggest that plasma 4-HNE may be a potential biomarker for stroke-related thrombosis, while further studies are needed to investigate the predictive role of 4-HNE on ischemic stroke.

Besides MDA and HNE, other lipid peroxidation biomarkers, including F2-isoprostanes (F2-IsoPs), oxidized LDL (oxLDL), and cholesteryl ester hydroperoxides (CEOOH), have also been investigated in stroke-related thrombosis. F2-IsoPs is a stable prostaglandin-like isomer biomarker of the oxidative stress status, which is produced by the oxidation of arachidonic acid mainly in membrane phospholipids [130]. In the study by Kelly et al., an increased level of F2-IsoPs was found in stroke patients compared with controls (median 0. 041 pg/mL versus 0.0295 pg/mL), indicating the close association between the F2-IsoPs level and stroke risk [131]. However, the application of F2-IsoPs as biomarkers of stroke is still highly limited in clinical settings due to high cost measurement, which requires gas/liquid chromatography coupled with mass spectroscopy techniques (HPLC/GC-MS). Moreover, the measurement of F2-IsoPs is likely to be inaccurate on account of the high frequency of arachidonic acid autoxidation if the sample has not been processed carefully [132]. Hence, these disadvantages should be considered when measuring F2-IsoPs in clinical situations. Interestingly, 8-iso-PGF2*α*, an isomer derived from F2-IsoPs, seems to be a more practical biomarker, which shows a higher specificity, stability, and sensitivity as the index of oxidative stress. Studies have observed that the level of urinary 8-iso-PGF2*α* is higher in stroke cases compared to controls [130]. Furthermore, when cardiovascular risk factors were adjusted, patients with higher concentration of urinary 8-iso-PGF2*α* had a greater risk of stroke (adjusted odds ratio 1.40) and urinary 8-iso-PGF2*α* could improve the discrimination between individuals with or without stroke [130]. These findings demonstrated that urinary 8-iso-PGF2*α* could be an independent lipid peroxidation biomarker for predicting the risk of stroke-related thrombosis.

Cholesteryl esters are one of the main components of LDL, and they can be easily oxidized into hydroperoxides, leading to the formation of CEOOH [133]. In the study by Polidori et al., stroke patients have been demonstrated to have higher concentrations of CEOOH compared to controls [134]. Moreover, the plasma CEOOH levels are positively associated with infarct volume [134], suggesting that the ischemic brain may contribute to CEOOH production, which can also be detected in the peripheral plasma. Therefore, plasma CEOOH may be an ideal biomarker to reflect the progression of thrombus formation in ischemic stroke and further studies are warranted to confirm this hypothesis.

Oxidized LDL (oxLDL) is another lipid peroxidation biomarker generated from LDL, and it has been found to disrupt endothelial function and promote platelet aggregation and leukocyte-endothelial cell adhesion [135, 136], thus playing a crucial role in the occurrence of stroke-related thrombosis. Indeed, there is a positive correlation between an increased plasma oxLDL level and acute ischemic stroke, suggesting that plasma oxLDL levels might be a potential indicator for stroke-related thrombosis formation [137]. To support this finding, Wang et al. found that in minor stroke or transient ischemic attack patients, higher serum oxLDL levels significantly increased the risk of recurrent stroke within 90 days [138, 139]. This result provided important evidence that higher serum oxLDL levels may indicate the progression of thrombosis development and clinicians should carefully monitor these patients. However, the application of oxLDL as a biomarker is under debate due to its low specificity, heterogeneity of oxidation products, and diverse results depending on the assay utilized [140].

### 4.2. DNA Oxidation Biomarkers

Oxidation of DNA components by oxidative stress is one of the major causes of DNA damage, which can result in nucleotide oxidation, loss of base, strand breakage, and adduct formation [141]. This reaction can generate various products, among which 8-hydroxy-2-deoxyguanosine (8-OHDG) is the most frequently studied biomarker on account of its relative abundance and high specificity [142]. Cumulative studies have shown that the 8-OHDG level is significantly increased in stroke patients compared with controls [143]. Moreover, in noncardioembolic stroke patients who are not treated with statins, higher peripheral levels of 8-OHDG are associated with stroke recurrence [144]. In conclusion, these studies have suggested that 8-OHDG may be an indicator of stroke-related thrombosis progression and close monitoring are required once the level of 8-OHDG is elevated after the first-ever ischemic stroke. However, it should be noted that the measurement of 8-OHDG has been impeded due to several technical drawbacks. Nowadays, although several methods are available for 8-OHDG detection, they are likely to measure oxidation artefacts [145], which largely decrease the accuracy. Thus, future studies are needed to solve this problem and identify a more accurate measurement method for 8-OHDG.

### 4.3. Antioxidants Biomarkers

As mentioned above, the disrupted redox balance caused by either excessive oxidant generation or insufficient antioxidant storage may lead to oxidative stress, which then participates in stroke-related thrombosis. Thus, the levels of antioxidants may also serve as indirect biomarkers of oxidative stress in the occurrence of stroke-related thrombosis.

Studies have confirmed that in patients with ischemic stroke, the levels of plasma nonenzymatic antioxidants vitamin C [146] and vitamin E [147] are significantly decreased compared to those in the healthy control group. In addition, there are strongly linear associations between blood concentrations of vitamin C or vitamin E and stroke-related thrombosis formation [148], and a higher serum level of vitamin C is associated with a decreased risk of ischemic stroke [149]. These studies provided evidence for the potential roles of vitamin C and vitamin E as biomarkers of stroke-related thrombosis. Additionally, UA is the final product of purine metabolism and is considered as the main antioxidant in the body by scavenging O_2_- and HO. In contrast to vitamin C and vitamin E, both the serum and saliva concentrations of UA were found to be increased in ischemic stroke patients [126]. Further study by Al-Rawi et al. showed that salivary UA can effectively differentiate ischemic stroke patients from healthy controls (accuracy 89.3%, AUC 0.95); however, the diagnostic utility of serum UA was significantly lower than salivary UA (accuracy 89.3%, AUC 0.927) [126]. Interestingly, UA concentration is also significantly elevated in patients with diabetes mellitus, hypertension, and ischemic heart disease, which are known risk factors of ischemic stroke [150, 151], and high UA level has been found to increase the risk of thrombosis-induced stroke in these patients [152–154]. In consideration of the fact that UA is a common and easily detected index in clinic, it may be a useful biomarker to assess the risk of stroke-related thrombosis formation.

With respect to the enzymatic antioxidants, there is very little or no concentration of CAT, but relatively low levels of SOD and GPXs in human serum [155]. Studies have shown that the concentrations of SOD and GPXs are altered in ischemic stroke patients. Moreover, GPXs are able to oxidize GSH to GSSG, resulting in a decreased level of GSH, which is considered to have critical clinical importance in stroke patients [156]. The result from the study by Al-Rawi et al. has shown that both serum (accuracy 80%, AUC 0.912) and salivary GSH (accuracy 81%, AUC 0.669) can significantly differentiate stroke patients from healthy individuals [126]. Therefore, GSH may be a potential antioxidant biomarker for the diagnosis of stroke-related thrombosis. High clinical utility of SOD has also been reported, and both serum (accuracy 80%, AUC 0.838) and saliva SOD (accuracy 89.3%, AUC 0.918) have been found to effectively differentiate between healthy and ischemic stroke individuals [126].

In conclusion, the changes in both nonenzymatic and enzymatic antioxidant levels shed light on predicting stroke-related thrombosis and monitoring its progression.

### 4.4. MPO Biomarkers

MPO plays an essential part in endothelial dysfunction as well as in platelets activation, indicating its potential role in stroke-related thrombosis. A recent study also investigated the association between MPO levels and stroke risk. Researchers found that genetic variants which could induce serum MPO concentration were able to increase the risk of lacunar stroke [157]. In addition, a case-cohort study, which enrolled 2176 participants and evaluated the correlation between 13 plasma biomarkers and stroke risk over a median follow-up of 5 years, proved that MPO was a potential biomarker that was independently associated with stroke risk [158].

### 4.5. Limitations and Gaps in the Study of Oxidative Stress Biomarkers in Stroke-Related Thrombosis

Currently, although a large number of studies have been aimed to discover oxidative stress biomarkers of stroke-related thrombosis, a clinically applicable biomarker remains elusive. There are many limitations and gaps which hinder their clinical use and we have summarized these drawbacks in the following paragraphs.

Firstly, methodological factors should be taken into consideration when measuring these biomarkers in clinical practice. An ideal clinical biomarker should possess the following characteristics: it should be easily and accurately measured, be stable and presented in an accessible specimen, and be cost-effective. However, most of these oxidative stress biomarkers can easily undergo autoxidation or react with another substance leading to inaccurate measurement, especially when the samples are not properly processed and stored. It seems that low-temperature storage and rapid separation of the biological sample using a refrigerated microcentrifuge may increase the stability of antioxidants and prevent thermal stress to some extent [159]. Additionally, the measurement of many oxidative stress biomarkers is largely based on high-performance liquid chromatography (HPLC), gas chromatography coupled with mass spectroscopy (GC-MS), or ELISA assays [160], which is highly expansive and difficult to popularize. These drawbacks in the measurement of oxidative stress biomarkers may partly explain their failure in clinical use and we are looking forward to novel methods to solve this problem.

Secondly, it should be noted that many confounding factors contribute to the impeded reliability of antioxidant measurement. In human studies, the production of antioxidants is actually influenced by a wide range of parameters including gender, age, vitamin supplementation, drug administration, alcohol consumption, nutritional status, physical activity, and smoking habit [161]. However, researchers find it very challenging to control these confounding factors in clinical studies and inconsistent baseline information of the subjects studied may contribute to variations in the antioxidant level.

Finally, most of the studies only measure one oxidative stress biomarker and investigate its single role in stroke-related thrombosis. Considering the complex and multifaceted process in the occurrence of oxidative stress, one biomarker may have relatively low sensitivity and specificity, which highly limits its clinical application. To solve this issue, it might be promising to combine more than one biomarker and establish a biomarker score system, which can contain all possible factors that can be reliably and simply tested. Currently, two score models, the OXY-SCORE [162] and oxidative-INDEX [163], have been proposed to comprehensively assess the status of oxidative stress and have shown powerful efficacy; however, their application in stroke-related thrombosis remains to be explored.

## 5. Antithrombotic Strategies in Ischemic Stroke for Oxidative Stress

In previous sections, we have highlighted that oxidative stress plays a crucial role in the pathogenesis of thrombus formation in ischemic stroke based on four aspects: endothelial dysfunction, platelet function, platelet-leukocyte aggregation, and fibrinogen modification. Significantly, the impact of oxidative stress may last for a long time once ischemic stroke occurs. Thus, targeting oxidative stress may help prevent the progression of stroke-related thrombus formation and in turn improve the prognosis of ischemic stroke. In the following section, we will discuss the potential role of antioxidants based on the following three aspects: free radical production inhibitors, free radical scavengers, and NO donor.

As mentioned above, the production of oxidative stress mainly relies on enzymes, such as NOX and XO, and drugs which can inhibit the activity of these enzymes may alleviate oxidative stress, exert antithrombotic effects, and prevent stroke. To support this, researchers has revealed that double inhibition of NOX and XO effectively prevented salt-induced stroke in spontaneously hypertensive rats [164], further elucidating that oxidative stress produced by NOX and XO can affect the susceptibility to stroke. Suppression of NOX with its nonselective inhibitor apocynin has been found to alleviate enhanced platelet activation [107] and endothelial cell dysfunction in aged mice [165], indicating its potential role in preventing the formation of thrombus. Additionally, treatment with apocynin has been shown to decrease the cerebral infarction size in experimental studies [166]. However, apocynin is neither specific nor selective to the seven NOX isoforms, while the different NOX subtypes may have opposite function in ischemic stroke [167, 168]. Thus, the application of apocynin is not appropriate and more specific NOX inhibitors, especially targeting NOX2 and NOX4 which are the main isoforms in thrombus formation, may be more effective in preventing stroke-related thrombosis. Specifically, the NOX2/4 inhibitor VAS2870 [169] was found to reduce platelet aggregation and delay thrombus formation via the PKC signaling pathway in experimental rodent models, while normal hemostasis was not affected by VAS2870 [170, 171], suggesting VAS2870 may be a potentially safe and effective agent for stroke-related thrombosis prevention. However, there is a lack of evidence from clinical trials regarding the role of VAS2870 in the prevention and treatment of ischemic stroke and further studies are warranted.

In addition to NOX inhibitors, XO inhibitors including allopurinol and febuxostat have also gained special attention in stroke-related thrombosis. In experimental studies, mice treated with the potent XO inhibitor febuxostat showed decreased stress-induced ROS generation, ameliorated endothelial dysfunction, and reversed prothrombotic state [172]. Notably, salt-induced thrombosis in spontaneously hypertensive rats can also be alleviated with febuxostat treatment [164]. These studies shed light on the role of febuxostat in preventing stroke-related thrombosis, while further large clinical trials are needed. In contrast to febuxostat, allopurinol seems to have no antithrombotic effect on free radical-induced thrombus formation [173, 174]. Consistent with this finding, randomized clinical trials on allopurinol treatment for stroke also failed and showed no improvement in cerebrovascular reactivity following ischemic stroke [175]. In conclusion, XO and NOX inhibitors have shown effects on preventing thrombosis in animal experiments, while further clinical studies are needed to test their effects on patients with high risk of stroke-related thrombosis formation.

Furthermore, researchers have also observed elevated MPO activity after ischemic stroke in basic research [176]. Both pharmacological MPO inhibition by 4-aminobenzoic acid hydrazide and congenital absence of MPO can decrease the final lesion volume and provide neuroprotection in rodent models of ischemic stroke [176, 177]. These studies highly imply the potentially beneficial role of MPO suppression in stroke-related thrombus progression. In one randomized, placebo-controlled, clinical trial in healthy humans, AZD4831, a selective and potent MPO inhibitor, showed considerable safety and tolerability, and effectively decreased UA concentrations after a single dose [178]. However, further studies are needed to assess the effect of AZD4831 in stroke-related thrombus prevention and progression.

Besides inhibition of ROS-producing enzymes, the application of compounds that are able to scavenge ROS may also be a promising strategy for preventing the occurrence and progression of stroke-related thrombosis. One of the most studied compounds is vitamin C, a potent radical scavenger and an antioxidant. In the basic experiment of occlusive aortic thrombus, the application of vitamin C to rats significantly reduced platelet aggregation and arterial superoxide generation, resulting in a delayed time to thrombus formation [179]. Moreover, a prospective cohort study has confirmed that a higher plasma vitamin C concentration is associated with reduced risk of ischemic stroke [180]. These studies highlighted the possibility of vitamin C as an antioxidant in suppressing stroke-related thrombosis. Considering that vitamin C is abundant in fruits and vegetables, it is worth studying whether the dietary intake of vitamin C has an impact on stroke-related thrombosis. A large number of prospective cohort studies on vitamin C have been performed in recent years. Disappointingly, almost all research studies concluded that vitamin C supplement cannot reduce the risk of stroke in healthy individuals [181–183], and only one study reported a positive result [184]. Consistent with this finding, a meta-analysis by Ye et al. which enrolled 15 trials also reported no significant effects on stroke prevention in the vitamin C intake group [185]. Different results have been obtained between experimental studies and clinical studies, and many factors may contribute to this discrepancy. Firstly, most cohort studies chose the dietary assessment to evaluate the vitamin C intake, which is an estimated and inaccurate indicator of the blood vitamin C concentration. Secondly, in experimental studies, the researchers were more likely to give a higher dose of vitamin C to animal models over a short time period. However, prospective cohort studies were designed to investigate the effect of long-term intake of low-dose dietary vitamin C on stroke prevention. Lastly, the contradictory results may have also resulted from participants' poor lifestyle habits, such as the high intake of salt and oil, in the low vitamin C intake group in clinical cohort studies. In conclusion, the design, study population, observation time, and endpoint of a clinical trial are essential to draw the conclusion of a clinical trial and well-designed trials are indispensable to gain a better understanding of the association between dietary intake of vitamin C and stroke-related thrombosis prevention.

Resveratrol, a natural polyphenolic antioxidant, has also been found to exert antithrombotic effects. In the basic research, resveratrol treatment has been found to enhance NOS activity and decrease NOX activity in platelets, showing antithrombotic activity [186]. Moreover, platelet aggregation induced by ADP, collagen, and thrombin was significantly inhibited by resveratrol treatment *in vitro* [187]. These studies highlighted the potential role of resveratrol in the prevention of stroke-related thrombosis. However, current evidence from large clinical trials is lacking and it will be interesting to investigate whether patients with high risk of stroke can benefit from resveratrol treatment. In addition, an increasing number of experimental research studies have demonstrated resveratrol-induced neuroprotection in the murine model of ischemic stroke by activating free radical scavenger PPAR*γ* coactivator 1*α* [188] and antioxidant enzyme HO-1 [189]. Significantly, in the clinical trials by Chen et al., administration of resveratrol has been found to improve functional outcomes in stroke patients receiving thrombolysis treatment [190]. However, it should be noted that a relatively small number of patients were included in the study and the researchers only evaluated the National Institute of Health Stroke Scale (NIHSS), lacking modified Rankin Scale (mRS) to assess the long-time functional improvement. Thus, further larger and well-designed RCTs are needed.

N-acetylcysteine (NAC), another free radical scavenger, has been clinically approved to regulate the redox status. In a mouse model of the prothrombotic type 1 diabetes, NAC treatment significantly ameliorated platelet activation and cerebral thrombosis [104]. Additionally, potent thrombolytic effect of NAC on arterial thrombus by proteolysis of vWF has also been observed [191]. However, the clinical trial evaluating the preventive role of NAC on stroke-related thrombosis is lacking. In addition, NAC treatment has been shown to improve the prognosis of patients with ischemic stroke. In two recent randomized clinical studies, oral NAC administration was positively associated with better functional and neurological outcomes in ischemic stroke patients [192, 193]. Nevertheless, it is worth noting that both studies enrolled a small number of patients from a single center and the therapy period was relatively short, which may influence the reliability of these studies. Thus, further trials that can address these issues are needed to fully evaluate the efficacy profile of NAC in stroke patients.

NXY-059 is another free radical spin-trap reagent and has received a lot of attention in both experimental and clinical studies of ischemic stroke. In a large number of preclinical rodents and primate species studies, NXY-059 has been shown to reduce infarction volume and improve functional outcomes [194–196], possibly by preventing the progression of stroke-related thrombosis. The promising results from the basic studies led to two large randomized clinical trials, Stroke-Acute Ischemic NXY Treatment (SAINT) I, and SAINT II trials. Although the SAINT I trial showed that NXY-059 significantly improved the mRS of ischemic stroke patients compared with the placebo group, an entirely negative result was reported in the subsequent SAINT II trial with a larger patient enrollment [197, 198]. The disparity in the outcomes of these two trials may be attributed to the inclusion criteria of patients, as more patients showed a poor latent prognosis in the SAINT I trial, as well as the statistical difference.

As mentioned above, NO has been shown to play a key role in the maintenance of endothelial function, thus preventing the progression of stroke-related thrombosis [55]. However, the vascular concentration of NO is decreased in ischemic stroke and has a close association with stroke severity and mortality [199]. Thus, it seems reasonable that NO supplementation may contribute to the prognosis of ischemic stroke and the prevention of second stroke. Indeed, the NO donor, glyceryl trinitrate, is currently in clinical trials for ischemic stroke, while the results from these randomized controlled trials are inconsistent [200–203]. A recent meta-analysis, which enrolled a total of 5363 patients, reported no effect on the 4-10 day mortality (relative risk, 1.11; 95% CI, 0.82-1.49) or 90 day mortality (relative risk, 0.96; 95% CI, 0.77-1.19) in ultraearly stroke patients (≤6 h) receiving glyceryl trinitrate treatment compared to placebo [204]. However, there were many limitations in this meta-analysis. The data analyzed in the study were mainly obtained from two trials, ENOS and RIGHT-2, which may have led to statistical bias; one research group conducted six out of the seven clinical trials; there were obvious heterogeneities in the patient clinical characteristics among the primary trials. Thus, a larger well-designed clinical trial is still needed to solve the problems.

Modulating oxidative stress may also help promote thrombolysis in acute ischemic stroke patients. At present, rt-PA, which contributes to fibrinolysis of thrombi, is the only clinical drug recommended by the FDA for intravenous thrombolysis in acute ischemic stroke patients within 4.5 hours after onset. However, even after intravenous injection of rt-PA, the successful arterial recanalization rate is at a relatively low level (about 20-46%) [205]. As discussed previously, oxidative stress can affect fibrinogen function and fibrin network formation, leading to prolonged lysis time of clots [17]. Thus, we speculated that the combination of antioxidant stress therapy and rt-PA may be helpful for thrombolytic therapy. Indeed, studies of antioxidant edaravone have shed light on this aspect. In the model of He-Ne-laser-induced thrombosis in mesenteric microvessels, the rt-PA combined with edaravone group showed significantly enhanced thrombolysis speed and reduced thrombus volume at 60 min after laser irradiation, compared with placebo and rt-PA groups [206]. This result suggested a possibility that the combination of rt-PA and edaravone can improve the efficacy of thrombolysis. Two possible mechanisms may explain this effect: firstly, previous studies have demonstrated that edaravone can elicit the release of NO from the endothelium to cause vasorelaxation and thus accelerate thrombolysis [207]; secondly, edaravone has been found to inhibit the expression of selectin, which participates in platelet adhesion, activation, aggregation and leukocyte adhesion [208]. Thus, this may impair new thrombus formation and facilitate thrombolysis. However, it should be noted that mesenteric microvessels may be different from central nervous system vessels and further studies are needed to confirm this finding.

Despite the success of antioxidative treatments in many experimental studies, most of the clinical trials have failed as summarized previously. The reason is unclear and many estimated factors may have contributed to this failure. Firstly, researchers tend to carry out experimental studies using young and male animals that have no comorbidities and this is inconsistent with the situation in clinical trials [209]. We know that ischemic stroke mostly occurs in old patients and they often suffer from more than one disease, which makes the treatment complicated. It might be better to evaluate the effects of antioxidants on old animals with no sex limitation. Secondly, a great number of experimental studies fail to follow rigid randomization and blinding in their study design and implementation [210], and this could have largely overestimated the efficacy. Thus, proper randomization and blinding are necessary to avoid subjective errors and improve the validity of studies. Finally, it is well known that the study design, duration of observation, primary endpoints, and study population are key factors for the outcome of one clinical trial. However, these factors are variable among different clinical trials and well-designed clinical studies are necessary to examine the efficacy. We also found that several antioxidants that achieved success in basic studies have not been investigated in clinical trials and we urge that further randomized studies should be conducted.

There is another limitation in current translational studies on the preventive role of antioxidants in stroke-related thrombosis. Considering the clinical trials investigating vitamin C, researchers preventively treated the whole enrolled population with vitamin C, rather than those with high levels of oxidative stress, to observe whether vitamin C intake can prevent ischemic stroke occurrence. Consequently, patients with low status of oxidative stress could suffer from adverse events, which may lessen the efficacy of antioxidants treatment and explain the failure. Thus, future clinical trials should focus on patients with high status of oxidative stress. This might be achieved by subgroup analysis in patients sorted out as the high-risk population according to previously mentioned oxidative stress biomarkers. This type of analysis might open up further options for patients to enroll in individualized preventive treatment schemes, which might be more accurate and effective.

It can be speculated that targeting oxidative stress to suppress stroke-related thrombosis will play an important role in clinical decision making. Currently, it remains largely unknown how to appropriately diminish oxidative stress with antioxidants and we expect further studies to overcome this problem.

## 6. Conclusion

In conclusion, oxidative stress contributes to the pathogenesis of stroke thrombus, and the possible mechanisms include disruption of endothelial function, activation of platelets, enhancement of platelet-leukocyte aggregation, and modification of fibrinogen. Positive feedback between oxidative stress and thrombosis usually exists in ischemic stroke patients. Moreover, there is a strong association between the concentration of oxidative stress biomarkers and the occurrence and progression of stroke-related thrombosis. In patients with a higher level of these biomarkers, the risk of developing stroke is significantly increased, and it is accompanied with poor clinical outcomes. Hence, targeting oxidative stress may be a novel strategy to prevent stroke occurrence and promote the dissolution of thrombus.

## Data Availability

No data were used to support this study.
